# Complete genome analysis of clinical *Shigella* strains reveals plasmid pSS1653 with resistance determinants: a triumph of hybrid approach

**DOI:** 10.1186/s13099-019-0334-5

**Published:** 2019-11-06

**Authors:** Dhiviya Prabaa Muthuirulandi Sethuvel, Balaji Veeraraghavan, Karthick Vasudevan, Naveen Kumar Devanga Ragupathi, Dhivya Murugan, Kamini Walia, Shalini Anandan

**Affiliations:** 1 Department of Clinical Microbiology, Christian Medical College, Vellore, Tamil Nadu 632004 India; 2 Division of Epidemiology and Communicable Diseases, Indian Council of Medical Research, New Delhi, 110 029 India

**Keywords:** Hybrid assembly, Pathogenicity island, Prophages, Insertion sequences, Plasmids

## Abstract

*Shigella* is ranked as the second leading cause of diarrheal disease worldwide. Though infection occurs in people of all ages, most of the disease burden constitutes among the children less than 5 years in low and middle income countries. Recent increasing incidence of drug resistant strains make this as a priority pathogen under the antimicrobial resistance surveillance by WHO. Despite this, only limited genomic studies on drug resistant *Shigella* exists. Here we report the first complete genome of clinical *S. flexneri* serotype 2a and *S. sonnei* strains using a hybrid approach of both long-read MinION (Oxford Nanopore Technologies) and short-read Ion Torrent 400 bp sequencing platforms. The utilization of this novel approach in the present study helped to identify the complete plasmid sequence of pSS1653 with structural genetic information of AMR genes such as *sul*II, *tet*A, *tet*R, *aph(6)*-*Id* and *aph(3′’)*-*Ib.* Identification of AMR genes in mobile elements in this human-restricted enteric pathogen is a potential threat for dissemination to other gut pathogens. The information on *Shigella* at genome level could help us to understand the genome dynamics of existing and emerging resistant clones.

## Introduction

*Shigella* is the second leading cause of diarrheal deaths globally, mainly among children less than 5 years. *Shigella flexneri* and *Shigella sonnei* are the leading cause of diarrhea in developing countries like India while other two serogroups are relatively uncommon [1]. Historically, *S. sonnei* is mainly seen in developed countries but its recent spread into developing countries over the last decades has raised major public health concerns [2]. Due to its low infectious dose, clinical severity, serotype specific immunity, emerging antimicrobial resistance and having humans as the only natural host, *Shigella* is categorized as a priority pathogen among enteric bacteria on Global Antimicrobial Resistance Surveillance System (GLASS) by World Health Organization (WHO) [3].

The key virulence factors that are involved in the pathogenesis of *Shigella* are located on both the plasmid and chromosome of the pathogen enabling it to survive intra-cellularly. Shigellosis is generally self-limiting but the use of antibiotics reduces the duration of symptoms and pathogen shedding which in turn reduces transmission. The increasing awareness of disease burden and emerging threats posed by drug resistant *Shigella* have resulted in an interest in the development of *Shigella* vaccines which are currently in the clinical trial stage [1].

There is an increasing interest in exploring the molecular epidemiology of genetically encoded virulence and resistance factors in *Shigella* as this provides information on the severity of infection, transmission and the pathogen response to antimicrobials. The virulence and resistance determinants are mainly located on mobile genetic elements (MGEs) such as plasmids, insertion sequences, integrons, pathogenicity islands and bacteriophages in *Shigella* spp. Horizontal gene transfer (HGT) of these elements acts as an important driver for bacterial evolution [4]. Through HGT, the pathogen enhance its ability to establish infection and to acquire resistance to outcompete other susceptible bacteria in the gut by transferring genes between the commensal and other pathogenic bacteria that are circulating locally [5, 6]. These MGEs can be predicted using whole genome sequencing (WGS) through bioinformatics analysis. Recently, the advancement of whole genome sequencing methodologies has a major impact on bacterial genoe wide studies and in the epidemiological analysis of bacterial pathogens.

In this study, we report the first complete genome of *S. flexneri* serotype 2a and *S. sonnei* strain using a hybrid assembly approach of both long-read MinION (Oxford Nanopore Technologies) and short-read Ion Torrent 400 bp sequencing platforms. The availability of the complete genome of *Shigella* clinical strains and subsequent genome analysis provides a better understanding into its genome characteristics including virulence, resistance and mobile genetic elements.

## Materials and methods

### Bacterial isolates

The two clinical *Shigella* strains, *S. flexneri* 2a (FC906) and *S. sonnei* (FC1653) sequenced were isolated from stool specimens at the Department of Clinical Microbiology, Christian Medical College, Vellore, India.

### Genome sequencing

Genomic DNA was extracted using QIAamp DNA Mini Kit (QIAGEN, Hilden, Germany) according to the manufacturer’s instructions. DNA quality and quantity was assessed using Nanodrop spectrophotometry (Thermofisher, USA) and Qubit 3.0 (Thermofisher, USA) respectively. To get the closed genome, a hybrid approach using long read MinION and short read IonTorrent sequencing was performed as described previously [7]. Briefly, short read sequencing was performed with 400-bp read chemistry using an IonTorrent™ Personal Genome Machine™ (PGM) (Life Technologies, Carlsbad, CA) as per manufacturer’s instructions. Long read sequencing was performed using SQK-LSK108 Kit R9 version (Oxford Nanopore Technologies, Oxford, UK) using 1D sequencing method according to manufacturer’s protocol.

### Assembly and annotation

The Fast5 files were generated from MinION sequencing and the reads were base called with Albacore 2.0.1 (https://nanoporetech.com/about-us/news/new-basecaller-now-performs-raw-basecalling-improved-sequencing-accuracy). Canu 1.7 was used for error correction of reads and assembly with genome size of 3.0 m as input [8]. The quality of the MinION reads was assessed using MinIONQC (https://github.com/roblanf/minion_qc). To increase the accuracy and completeness of genome, we performed hybrid assembly using both Ion torrent and MinION reads with Unicycler (v0.4.7) [9]. By default, unicycler utilizes SPAdes [10] to assemble the short reads with different k-mers and filter out the low depth regions along with error correction and quality checks. Subsequently, it trims and generates the short read assembly graph. In addition, it uses Miniasm [11] and Racon [12] to assemble the MinION long reads and further the reads were bridged to determine all the genome repeats and produces complete genome assembly. In addition, multiple rounds of short reads polishing was performed with Pilon [13] to reduce the base level errors in long read assembly.

After assembly, the genomes were annotated using the NCBI Prokaryotic Genome Annotation Pipeline (PGAP). Virulence and antimicrobial resistance genes (ARG) were detected in silico by VirulenceFinder ((https://cge.cbs.dtu.dk/services/VirulenceFinder/) [14] and ResFinder (https://cge.cbs.dtu.dk/services/ResFinder/) database respectively with the 90% threshold for identity and with 60% of minimum length coverage [15]. Sequence type of the isolates were analyzed using MLST 2.0 (Multi Locus Sequence Typing) tool (https://cge.cbs.dtu.dk//services/MLST/) [16]. *Shigella* PAIs was compared with the reference sequences through BLASTn and visualized using Easyfig [17]. The genomes were screened for prophages using PHAST tool [18]. ISsaga was used to predict the number of insertion sequences in the genome (https://www-issaga.biotoul.fr/issaga_index.php) [19].

### Quality assurance

Species confirmation was performed by biochemical tests (motility, urea, citrate, indole, triple sugar iron) and species specific PCR was done [20, 21]. A pure isolated colony was used for genomic DNA extraction. The strain identification was confirmed through BLAST annotation using NCBI database and species was predicted using KmerFinder available at center for genomic epidemiology.

## Results and discussion

### Genome features

**Fig. 1:**
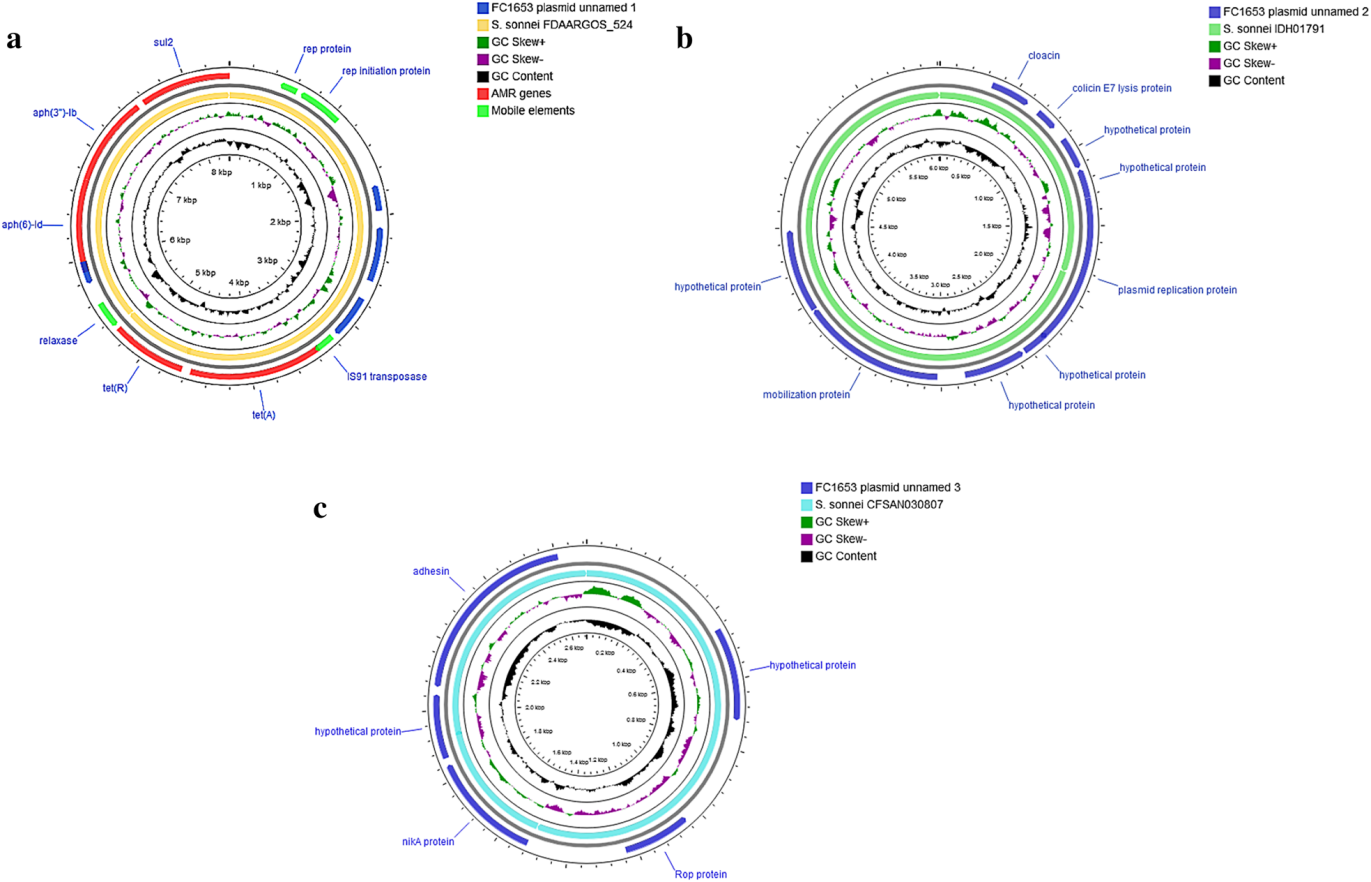
**a** Circular representation of unnamed plasmid 1, pSS1653 carrying AMR genes identified in *S. sonnei* (red color indicates AMR genes, green color indicates mobile elements, other CDS shown in blue color). The direction of arrows indicates the orientation of open reading frames (ORFs). **b** Circular representation of genetic arrangement of unnamed plasmid 2 identified in *S. sonnei* (blue and green color denotes CDS and reference sequence respectively). The direction of arrows indicates the orientation of open reading frames (ORFs). **c** Circular representation of genetic arrangement of unnamed plasmid 2 identified in *S. sonnei* (dark blue and light blue color denotes CDS and reference sequence respectively). The direction of arrows indicates the orientation of open reading frames (ORFs)

A hybrid assembly approach provided a complete single chromosome for *S. flexneri* (FC906) as well as chromosome and 3 plasmids with size of 8401 bp, 6015 bp and 2690 bp for *S. sonnei* (FC1653). On BLAST analysis, the plasmids showed 100%, 99.7% and 100% similarity against previously identified plasmids *S. sonnei* FDAARGOS_524 plasmid unnamed2, *S. sonnei* IDH01791 plasmid pSSE3 and *S. sonnei* CFSAN030807 plasmid pCFSAN030807_8 respectively. The comparison of genetic content of the plasmids against its respective reference plasmid are depicted in Fig. 1a–c. Utilization of this approach facilitates the complete genome analysis of clinical strains, especially in studying the structural arrangement of mobile genetic elements which plays a major role in AMR dissemination. The genome features of the sequenced isolates are given in the Table 1.

**Table 1:** Genomic features and Predicted insertion sequence elements of *S. flexneri* (FC906) and *S. sonnei* (FC1653) by hybrid assembly approach

	*S. flexneri* (FC906)	*S. sonnei* (FC1653)
Genomic features		
Length	4,655,489 bp	4,807,231 bp
Coverage	278×	174×
Contigs	1 (chromosome)	4 (1 chromosome, 3 plasmid)
Total genes	4770	4932
Total CDS	4637	4806
Total pseudo genes	735	857
tRNAs	104	95
rRNAs	22	22
GC content	50.8	51.0
Virulence factor (VFDB)	66	57
Resistance (CARD)	90	86
Sequence type	245	152

IS family	ORFs distribution^a^	Different IS	Total IS	ORFs distribution^a^	Different IS	Total IS
Insertion sequence elements predicted using ISsaga						
IS4_ssgr_IS4	[2/42/0/0]	1	44	[1/85/0/0]	1	86
ISL3	[0/1/0/3]	4	4	[0/0/0/1]	1	1
ISNCY_ssgr_ISPlu15	[4/0/0/0]	4	4	[2/1/0/2]	5	5
IS4_ssgr_IS50	[0/0/0/1]	1	1	[0/0/0/1]	1	1
IS3_ssgr_IS3	[45/126/0/0]	4	105	[26/132/0/1]	1	94
IS1	[107/14/0/0]	2	114	[119/60/0/0]	2	172
IS66	[6/22/0/0]	2	14	[3/3/0/0]	1	2
IS3_ssgr_IS150	[2/3/0/0]	1	3	[0/1/0/0]	1	1
IS200_IS605	[0/6/0/0]	2	6	[0/4/0/0]	2	4
IS91	[0/11/0/0]	3	11	[1/0/0/0]	1	1
IS3_ssgr_IS2	[22/52/0/0]	1	44	[26/46/0/0]	2	45
IS3_ssgr_IS51	[19/31/0/1]	2	32	[5/6/0/1]	2	7
IS110_ssgr_IS1111	[1/0/0/0]	1	1	[5/11/0/0]	2	16
IS110	[3/2/0/0]	1	5	[1/13/0/0]	1	14
ISAs1	–	–	–	[2/5/0/0]	1	7
IS21	–	–	–	[13/68/0/0]	2	50
IS630	–	–	–	[1/28/0/0]	1	29
IS256	[0/1/0/0]	1	1	–	–	–
Tn3	[1/0/0/0]	1	1	–	–	–
IS4_ssgr_IS10	[1/0/0/0]	1	1	–	–	–

^a^Complete/partial/pseudogene/unknown

The annotated chromosome of FC906 has been deposited in GenBank under accession number CP037996. For FC1653, the annotated chromosome and plasmids have been deposited under accession numbers CP037997 and CP037998, CP037999, CP038000, respectively.

### Virulence and resistance determinants

The *S. flexneri* genome possesses virulence genes such as invasion plasmid antigen (*ipa*H), long polar fimbriae (*lpf*A), and serine protease autotransporter protein (*pic* and *sig*A) belongs to SPATEs family. Alike, *S. sonnei* genome carried invasion plasmid antigen (*ipa*H), long polar fimbriae (*lpf*A), enterotoxin ShET-2 (*sen*B) and serine protease autotransporter protein (*sig*A). Generally the *ipa*H family genes are present in multiple copies on both the virulence plasmid and chromosome of the *Shigella* genomes [22]. However, the gene was identified in chromosome in the sequenced isolates.

Further, the toxin genes that belongs to SPATE family has been commonly categorized into 2 classes. The gene *sig*A belongs to class 1 and are toxic to epithelial cells, whereas *pic* gene is non-toxic and usually involved in colonization. These were first reported in *S. flexneri* serotype 2a which is in accordance with the present study [23]. In addition, the gene encoding *Shigella* enterotoxin 2 identified in *S. sonnei*, is reported to be involved in invasion process and play an important role in transport of electrolytes [24].

The genomes were also found to contain multiple resistance genes conferring resistant to streptomycin, beta-lactamase, tetracycline, trimethoprim/sulfamethoxazole, aminoglycosides and chloramphenicol. Resistance genes such as *aad*A1, *bla*_OXA-1_, *tet*B, *dfr*A1, and *cat*A1 were identified in the *S. flexneri* chromosome. In *S. sonnei*, *dfr*A1 gene was identified in chromosome, the genes *sul*II, *aph(6)*-*Id*, *aph(3’’)*-*Ib* and *tet*(A) were identified in plasmid 1, herein named as pSS1653. These were the acquired resistance genes commonly reported among *Shigella* spp. On mutation analysis in quinolone resistance determining region (QRDR), *S. flexneri* had double mutations in *gyr*A (S83L and D87N) and single mutation in *par*C (S80I) genes. Similarly, *S. sonnei* had mutations S83L and D87G in *gyr*A and S80I in *par*C genes. No mutations were observed in *gyr*B gene. These mutations are commonly associated with fluroquinolone resistance in *Shigella* spp. as reported in previous studies [25–27].

### Mobile genetic elements and pathogenicity island

Mobile elements such as bacteriophages, integrons, IS elements and PAIs are the major drivers of *Shigella* genome evolution and plasticity. They play a crucial role in pathogen virulence and in resistance spread. Analysis revealed the presence of class 1 integrons in *S. flexneri* and no integron in *S. sonnei*. In addition, the insertion sequences (IS) elements in *Shigella* are found to contribute to the antibiotic resistance and the evolution of the pathogen [28]. *Shigella* genomes naturally harbour hundreds of IS and inactivation of genes (formation of pseudogenes) have been caused by IS, either through IS mediated interruption or IS mediated genome rearrangement. This inactivation of genes hinders the ability of *Shigella* to cause disease in humans [28, 29]. In this study, 735 and 857 pseudogenes were identified in *S. flexneri* and *S. sonnei* respectively. Also a total of 391 and 535 IS elements were predicted to be present in *S. flexneri* and *S. sonnei* genomes. The most common family identified in both the genome was the IS1 family, accounting for approximately 29% and 32% of the IS elements, followed by IS3_ssgr_IS3 family in *S. flexneri* and *S. sonnei*. The predicted IS elements were given in Table 1.

In *Shigella*, the serotype conversion is generally mediated by bacteriophages [30]. The hybrid assembly analysis revealed, 15 phage regions (8 intact, 4 incomplete, 3 questionable) in *S. flexneri*. Similarly in *S. sonnei*, 15 phage regions with 5 intact, 6 incomplete and 4 questionable were identified. The phage regions covers approximately 10% and 7% of the entire chromosome of *S. flexneri* and *S. sonnei* respectively. On the third phage region of the *S. flexneri* chromosome, intact SfII bacteriophage was identified which is responsible for conferring the serotype 2a. The details of the identified prophages, length, position, number of CDS and GC content are provided in Tables 2 and 3.

**Table 2:** Prophage content of *S. flexneri* (FC906) analyzed using PHAST tool

Region	Length (Kb)	CDS	GC content (%)	Position	Completeness^a^	Possible phages^b^	Accession number
1	22.5	9	45.02	609,158–631,696	Incomplete	Bacillus_Blue (3)	NC_031056
2	19	19	51.73	1,558,706–1,577,795	Intact	Enterobacteria_UAB_Phi20 (10)	NC_031019
3	35.4	30	47.11	1,631,678–1,667,092	Intact	Shigella_SfII (11)	NC_021857
4	70	64	51.98	2,016,293–2,086,335	Intact	Phage_Gifsy_1 (15)	NC_010392
5	46.6	38	49.23	2,213,449–2,260,088	Intact	Salmonella_118970_sal3 (5)	NC_031940
6	36.1	27	48.89	2,408,721–2,444,902	Incomplete	Salmonella_SJ46 (2)	NC_031129
7	15.7	24	50.80	2,540,849–2,556,550	Questionable	Geobac_E2 (3)	NC_009552
8	55.4	67	49.55	2,749,711–2,805,160	Intact	Salmonella_SJ46 (7)	NC_031129
9	14	22	48.79	2,947,521–2,961,586	Incomplete	Enterobacteria_933 W (3)	NC_000924
10	6.9	7	49.11	3,195,314–3,202,254	Incomplete	Shigella_SfIV (1)	NC_022749
11	30.9	43	52.60	3,290,710–3,321,696	Intact	Enterobacteria_mEp460 (13)	NC_019716
12	29.9	26	47.50	3,416,333–3,446,293	Questionable	Shigella_SfII (3)	NC_021857
13	17.5	27	51.59	3,449,797–3,467,310	Intact	Stx2_c_1717 (6)	NC_011357
14	44.5	27	51.58	3,573,186–3,617,688	Intact	Enterobacteria_P1 (3)	NC_005856
15	9.6	11	50.59	4,048,439–4,058,064	Questionable	Aeromonas_vB_AsaM_56 (4)	NC_019527

^a^Prediction of prophage region, intact (score > 90), questionable (score 70–90) or incomplete (score < 70)

^b^Phage with the highest number of proteins most similar to those in the region

**Table 3:** Prophage content of *S. sonnei* (FC1653) analyzed using PHAST tool

Region	Length (Kb)	CDS	GC content (%)	Position	Completeness^a^	Possible phages^b^	Accession number
1	31.9	44	50.44	2,093,301–2,125,236	Intact	Aggregatibacter_S1249 (4)	NC_013597
2	16	10	51.97	2,127,662–2,143,700	Questionable	Escherichia_Av_05 (2)	NC_025830
3	13.7	19	49.02	2,656,501–2,670,224	Questionable	Enterobacteria_phiP27 (4)	NC_003356
4	9.9	9	50.68	2,850,233–2,860,210	Incomplete	Bacillus_G (2)	NC_023719
5	14.7	21	48.72	2,985,856–3,000,602	Incomplete	Shigella_Ss_VASD (3)	NC_028685
6	24.8	33	51.37	3,194,522–3,219,407	Intact	Enterobacteria_mEp460 (6)	NC_019716
7	48.7	52	48.90	3,303,427–3,352,149	Intact	Salmonella_BPS15Q2 (7)	NC_031939
8	22.3	12	51.21	3,615,693–3,638,085	Incomplete	Escherichia_Av_05 (2)	NC_025830
9	14.4	13	50.42	3,748,403–3,762,850	Incomplete	Cronobacter_vB_CsaM_GAP32 (2)	NC_019401
10	54.6	66	50.87	3,872,085–3,926,723	Intact	Enterobacteria_lambda (20)	NC_001416
11	10.4	12	50.08	4,139,568–4,149,969	Questionable	Stx2_c_1717 (3)	NC_011357
12	26.9	30	51.76	4,213,478–4,240,403	Intact	Enterobacteria_phiP27 (13)	NC_003356
13	19	24	49.54	4,260,910–4,279,909	Questionable	Enterobacteria_SfI (7)	NC_027339
14	9.5	11	50.95	4,294,012–4,303,528	Incomplete	Gordon_OneUp (2)	NC_030917
15	7.9	10	49.00	4,404,915–4,412,855	Incomplete	Shigella_Sf6 (1)	NC_005344

^a^Prediction of prophage region, intact (score > 90), questionable (score 70–90) or incomplete (score < 70)

^b^Phage with the highest number of proteins most similar to those in the region

Pathogenicity islands are the clusters of mobile elements that encode various virulence factors [30]. PAI such as SHI-1 (also called *she*), SHI-2 and *Shigella* resistance locus (SRL) were identified in *S. flexneri* genome. SHI-1 contains virulence genes like *pic* and *sig*A. SHI-2 comprising of genes encoding a aerobactin operon, iron acquisition siderophore system, transposases and several hypothetical proteins that are associated with the increased virulence of the pathogen [30]. The resistance locus, SRL contains *aad*A1, *bla*_OXA-1_, *cat* and *tet* genes conferring resistance to streptomycin, beta-lactams, chloramphenicol and tetracyclines.

**Fig. 2:**
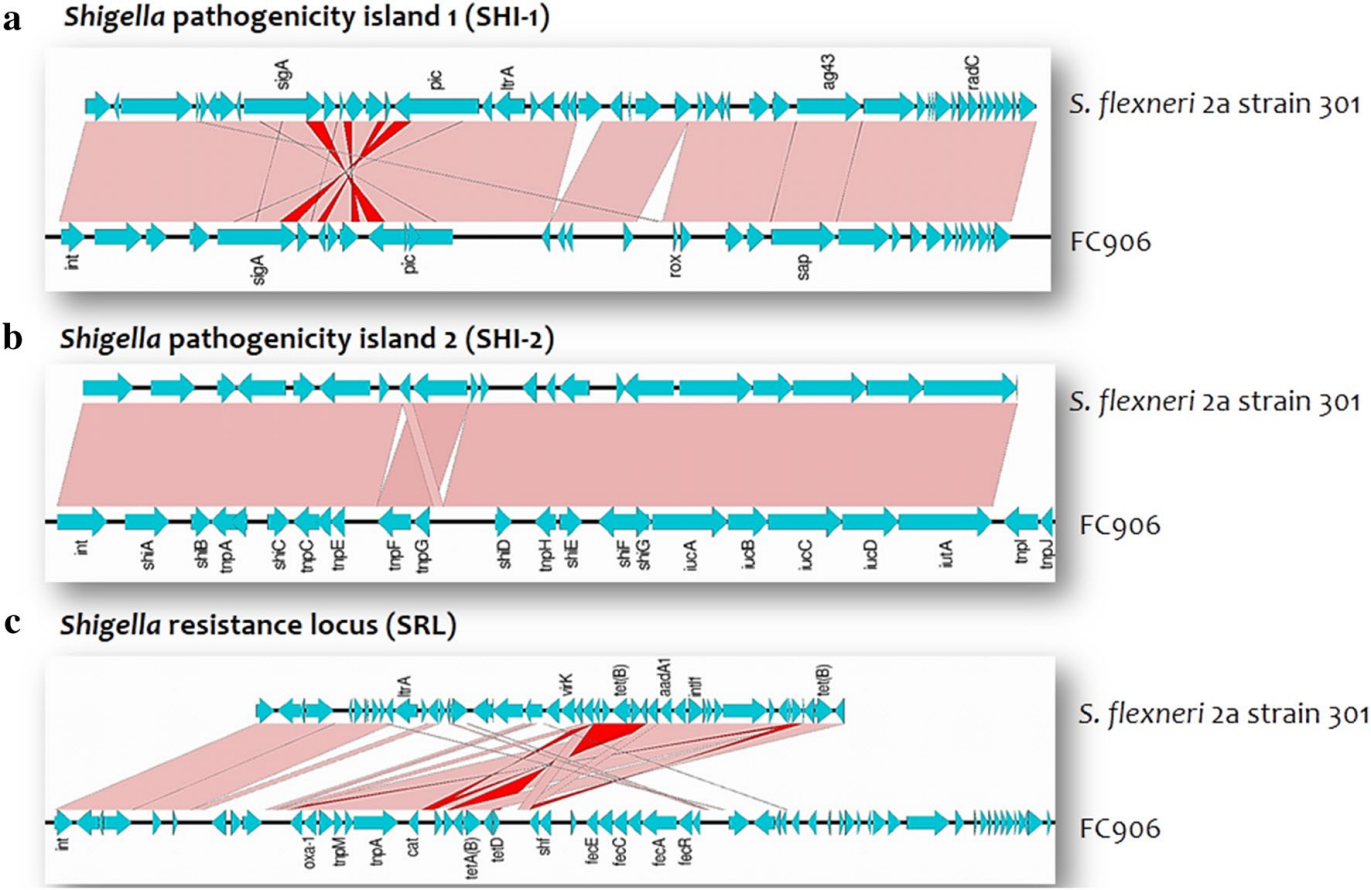
BLAST comparison of *Shigella* pathogenicity islands identified in *S. flexneri* (FC906) against reference sequence using Easyfig. **a** SHI-1 pathogenicity island, **b** SHI-2 pathogenicity island, **c** *Shigella* resistance locus (SRL) carrying antimicrobial resistance genes. Vertical blocks between the two sequences indicate the shared similarity regions shaded according to BLASTn (the pink shading indicate the matches in the same direction and red for inverted matches)

**Fig. 3:**
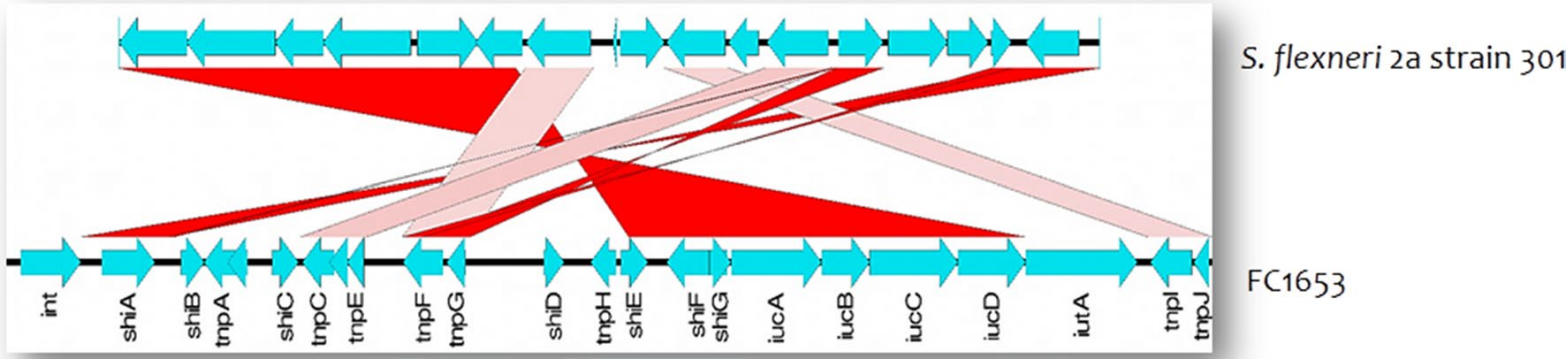
BLAST comparison of *Shigella* pathogenicity island (SHI-2) identified in *S. sonnei* against reference sequence using Easyfig. Vertical blocks between the two sequences indicate the shared similarity regions shaded according to BLASTn (the pink shading indicate the matches in the same direction and red for inverted matches)

Whereas, SHI-1 was absent in *S. sonnei*, and possess only SHI-2 island. This could be due to the ability of the SHI-1 to undergo spontaneous and specific excision via site-specific recombination [31]. This shows that *S. sonnei* might have lost its SHI-1 region in the course of evolution process to add other important genes for their successful survival. These pathogenicity islands are reported to be associated with phage integrases, suggesting the role of phages in the evolution of *Shigella* [32]. The BLAST comparison of these islands with reference was shown in Figs. 2 and 3.

The present study provided insights into the genetic content and complete structure of various mobile genetic elements that carries virulence and resistance determinants. Though, whole genome sequencing is a valuable tool for studying the bacterial genomes, the short read assembly (IonTorrent) could provide only limited information, particularly on the complete mobile genetic elements. However, long read assembly (MinION) could generate closed genome with enhanced information on the structural arrangement of mobile elements but with high error rate. Interestingly, the hybrid assembly approach involving short and long reads provided complete genome with acceptable error rate (< 10%). Thus the utilization of this novel approach in the present study helped to identify the complete plasmid sequence of pSS1653 with structural genetic information of AMR genes such as *sul*II, *tet*A, *tet*R, *aph(6)*-*Id* and *aph(3’’)*-*Ib.* Identification of AMR genes in mobile elements in this human-restricted enteric pathogen is a potential threat for dissemination to other gut pathogens. Further, limited information available on *Shigella* at genome level calls for a genomic surveillance studies to monitor the evolutionary trends and genome dynamics of emerging and existing resistance clones.

## Data Availability

Not applicable.

## References

[1] Kotloff KL, Riddle MS, Platts-Mills JA, Pavlinac P, Zaidi AKM (2018). Shigellosis. Lancet..

[2] Thompson CN, Duy PT, Baker S (2015). The rising dominance of Shigella sonnei: an intercontinental shift in the etiology of bacillary dysentery. PLoS Negl Trop Dis..

[3] World Health Organization. Global antimicrobial resistance surveillance system (GLASS) report: early implementation 2017–2018.

[4] Juhas M (2015). Horizontal gene transfer in human pathogens. Crit Rev Microbiol.

[5] Ragupathi NK, Sethuvel DP, Gajendran R, Anandan S, Walia K, Veeraraghavan B (2019). Horizontal transfer of antimicrobial resistance determinants among enteric pathogens through bacterial conjugation. Curr Microbial..

[6] Holt KE, Nga TV, Thanh DP, Vinh H, Kim DW, Tra MP, Campbell JI, Hoang NV, Vinh NT, Van Minh P, Thuy CT (2013). Tracking the establishment of local endemic populations of an emergent enteric pathogen. Proc Natl Acad Sci.

[7] Vasudevan K, Ragupathi NK, Jacob JJ, Veeraraghavan B (2019). Highly accurate-single chromosomal complete genomes using IonTorrent and MinION sequencing of clinical pathogens. Genomics.

[8] Koren S, Walenz BP, Berlin K, Miller JR, Phillippy AM (2017). Canu: scalable and accurate long-read assembly via adaptive k-mer weighting and repeat separation. Genome Res.

[9] Wick RR, Judd LM, Gorrie CL, Holt KE (2017). Unicycler: resolving bacterial genome assemblies from short and long sequencing reads. PLoS Comput Biol.

[10] Bankevich A, Nurk S, Antipov D, Gurevich AA, Dvorkin M, Kulikov AS, Lesin VM, Nikolenko SI, Pham S, Prjibelski AD, Pyshkin AV, Sirotkin AV, Vyahhi N, Tesler G, Alekseyev MA, Pevzner PA (2012). SPAdes: a new genome assembly algorithm and its applications to single-cell sequencing. J Comput Biol.

[11] Li H (2016). Minimap and miniasm: fast mapping and de novo assembly for noisy long sequences. Bioinformatics.

[12] Vaser R, Sović I, Nagarajan N, Šikić M (2017). Fast and accurate de novo genome assembly from long uncorrected reads. Genome Res.

[13] Walker BJ, Abeel T, Shea T, Priest M, Abouelliel A, Sakthikumar S, Cuomo CA, Zeng Q, Wortman J, Young SK, Earl AM (2014). Pilon: an integrated tool for comprehensive microbial variant detection and genome assembly improvement. PLoS ONE.

[14] Joensen KG, Scheutz F, Lund O, Hasman H, Kaas RS, Nielsen EM (2014). Real-time whole genome sequencing for routine typing, surveillance, and outbreak detection of verotoxigenic Escherichia coli. J Clin Microbiol.

[15] Zankari E, Hasman H, Cosentino S, Vestergaard M, Rasmussen S, Lund O (2012). Identification of acquired antimicrobial resistance genes. J Antimicrob Chemother.

[16] Larsen MV, Cosentino S, Rasmussen S, Friis C, Hasman H, Marvig RL (2012). Multilocus sequence typing of total genome sequenced bacteria. J Clin Microbiol.

[17] Sullivan MJ, Petty NK, Beatson SA (2011). Easyfig: a genome comparison visualizer. Bioinformatics.

[18] Zhou Y, Liang Y, Lynch KH, Dennis JJ, Wishart DS (2011). PHAST: a fast phage search tool. Nucleic Acids Res.

[19] Varani AM, Siguier P, Gourbeyre E, Charneau V, Chandler M (2011). ISsaga is an ensemble of web-based methods for high throughput identification and semi-automatic annotation of insertion sequences in prokaryotic genomes. Genome Biol.

[20] Bopp CA, Brenner FW, Fields PL, Murray PR, Baron EJ, Jorgensen J, Pfaller MA, Yolken RH (2003). Escherichia, Shigella, and Salmonella. Manual of clinical microbiology.

[21] Kim HJ, Ryu JO, Song JY, Kim HY (2017). Multiplex polymerase chain reaction for identification of shigellae and four Shigella species using novel genetic markers screened by comparative genomics. Foodborne Pathog Dis..

[22] Venkatesan MM, Buysse JM, Kopecko DJ (1989). Use of Shigella flexneri ipaC and ipaH gene sequences for the general identification of *Shigella* spp. and enteroinvasive *Escherichia coli*. J Clin Microbiol.

[23] Nave HH, Mansouri S, Moghadam MT, Moradi M (2016). Virulence gene profile and multilocus variable-number tandem-repeat analysis (MLVA) of enteroinvasive *Escherichia coli* (EIEC) isolates from patients with diarrhea in Kerman, Iran. Jundishapur J Microbiol..

[24] Zaidi MB, Estrada-García T (2014). Shigella: a highly virulent and elusive pathogen. Curr Trop Med Rep..

[25] Zhu Z, Cao M, Zhou X, Li B, Zhang J (2017). Epidemic characterization and molecular genotyping of *Shigella flexneri* isolated from calves with diarrhea in Northwest China. Antimicrob Resist Infect..

[26] Gu B, Qin TT, Fan WT, Bi RR, Chen Y, Li Y, Ma P (2017). Novel mutations in gyrA and parC among *Shigella sonnei* strains from Jiangsu Province of China, 2002–2011. Int J Infect Dis..

[27] Cui X, Wang J, Yang C, Liang B, Ma Q, Yi S, Li H, Liu H, Li P, Wu Z, Xie J, Jia L, Hao R, Wang L, Hua Y, Qiu S, Song H (2015). Prevalence and antimicrobial resistance of Shigella flexneri serotype 2 variant in China. Front Microbiol..

[28] Prosseda G (2012). Shedding of genes that interfere with the pathogenic lifestyle: the Shigella model. Res Microbiol.

[29] Wei J (2003). Complete genome sequence and comparative genomics of *Shigella flexneri* serotype 2a strain 2457T. Infect Immun.

[30] Parajuli P, Deimel LP, Verma NK (2019). Genome analysis of Shigella flexneri serotype 3b strain SFL1520 reveals significant horizontal gene acquisitions including a multidrug resistance cassette. Genome Biol Evol..

[31] Sakellaris H, Luck SN, Al-Hasani K, Rajakumar K, Turner SA, Adler B (2004). Regulated site-specific recombination of the she pathogenicity island of *Shigella flexneri*. Mol Microbiol.

[32] Ingersoll M, Groisman EA, Zychlinsky A, Hacker J, Kaper JB (2002). Pathogenicity Islands of Shigella. Pathogenicity islands and the evolution of pathogenic microbes. Current topics in microbiology and immunology, vol 264/2.

